# Process Hygiene Criterion for *Campylobacter* and Number of *Campylobacter* Enteritis Cases in Northwest Germany

**DOI:** 10.3390/foods13020281

**Published:** 2024-01-16

**Authors:** Tobias Nolte, Fabian Spieß, Anne-Katrin Jacobs, Nicole Kemper, Christian Visscher

**Affiliations:** 1 Science and Innovation for Sustainable Poultry Production (WING), University of Veterinary Medicine Hannover, Foundation, D-49377 Vechta, Germany; anne-katrin.jacobs@tiho-hannover.de (A.-K.J.); nicole.kemper@tiho-hannover.de (N.K.); christian.visscher@tiho-hannover.de (C.V.); 2 Institute for Animal Nutrition, University of Veterinary Medicine Hannover, Foundation, D-30173 Hannover, Germany; fabian.spiess@tiho-hannover.de; 3 Institute for Animal Hygiene, Animal Welfare and Farm Animal Behaviour, University of Veterinary Medicine Hannover, Foundation, D-30173 Hannover, Germany

**Keywords:** broiler, slaughter hygiene, process hygiene criterion, *Campylobacter*, foodborne pathogen, one health, zoonosis

## Abstract

Campylobacteriosis is the most commonly reported bacterial foodborne disease in the European Union. Its transmission is often associated with the consumption of poultry meat. In 2018, Regulation (EC) No. 2017/1495 introduced a process hygiene criterion and with this, the testing requirements for *Campylobacter*. The results of microbiological testing for *Campylobacter* of chicken carcass neck skin samples from several slaughter lines in Northwest Germany collected by the food business operators and contamination levels (cfu/g *Campylobacter*) of these samples were analysed from 2018 to 2021. Classification into three different categories was made based on contamination levels. The proportion of highly contaminated (category three) neck samples (>1000 cfu/g) decreased from 2018 to 2021. Our analysis showed a relationship between the number of neck samples with high *Campylobacter* contamination levels (>1000 cfu/g) and human cases in Northwest Germany. Spearman’s rank test (*p* < 0.01) showed a higher correlation in 2018 (0.66) and 2019 (0.58) compared to 2020 and 2021. *Campylobacter* enteritis cases in Northwest Germany stayed at a low level in 2020 and 2021. It remains unclear whether the decrease in reported *Campylobacter* enteritis cases is related to a decrease in *Campylobacter* levels on chicken carcasses or due to other reasons like underreporting during the COVID-19 pandemic, and therefore must be investigated in further analyses.

## 1. Introduction

*Campylobacter* enteritis is the most common foodborne diarrhoeal disease in humans in Germany, with more than 67,000 reported cases in 2018. Furthermore, it is also one of the bacterial diseases most frequently reported to the European Centre for Disease Prevention and Control (ECDC) throughout the European Union (EU), with more than 246,000 cases [1,2]. Due to a high number of unreported cases, the number of cases in the EU is estimated to be 9 million cases annually [3]. The treatment costs associated with the disease are around EUR 2.4 billion per year throughout the EU [3]. With an increased number of cases in the summer months and a lower number of cases in the winter months, there is a well-known seasonality [4,5]. The bacterial species *Campylobacter jejuni* is responsible for the most *Campylobacter* infections in humans, followed by infections caused by *Campylobacter coli* [5]. The typical symptoms of the disease are diarrhoea, abdominal pain or cramps, fever and lassitude. The duration of the disease is usually one week. In rare cases, secondary diseases may occur. These include reactive arthritis, irritable bowel syndrome and neurological complications such as the Guillain–Barre syndrome [6]. Children under five years of age and young adults between 20 and 29 years of age are most commonly affected [6].

As a zoonotic pathogen, which causes the reciprocal transmission of diseases between humans and animals, *Campylobacter* in general originates predominantly from livestock. Overall, between 50 and 80% of *Campylobacter* enteritis cases can be attributed to chicken flocks as a whole [7]. The Robert Koch Institute (RKI) identified the consumption of chicken meat and eating out as the greatest risk factors for human infection with *Campylobacter* in Germany [6]. According to the European Food Safety Authority (EFSA), mistakes in handling, preparation and consumption of broiler meat account for 20–30% of *Campylobacter* enteritis cases [7]. In addition to the consumption of chicken meat and eating out, raw milk, contaminated drinking water and surface water as well as international travel are other risk factors [8,9].

In primary production, there are different risk factors leading to the introduction of *Campylobacter* into a poultry flock. These include poor biosecurity, flock thinning, contaminated drinking water and increased animal age [3,10,11,12]. Once *Campylobacter* has entered the flock, the spread of *Campylobacter* within the flock is rapid and the prevalence can increase from 5% to 95% within one week [13]. The flock prevalence of *Campylobacter* of up to 70% in German livestock has been reported before [14]. Animals infected with *Campylobacter* often excrete faeces asymptomatically, i.e., without showing signs of disease, and thus can be a source of infection [15].

Since the EFSA recommended a number of control measures for the containment of *Campylobacter* along the process chain of poultry meat production, it has been obligatory for German slaughterhouses to examine *Campylobacter* in broiler chickens within the process hygiene criterion (PHC) for *Campylobacter* in accordance with Regulation (EC) No. 2017/1495 since 1 January 2018 [16]. The PHC for *Campylobacter* has brought the slaughtering process into greater focus as there are critical points during the slaughtering process that can lead to the *Campylobacter* contamination of broiler carcasses [17]. Thus, previous studies described an increase in *Campylobacter* concentration after defeathering and evisceration, whereas lower detection rates were observed after scalding [18,19,20]. The purpose of the PHC is the assumption that the risk to public health from the consumption of broiler meat can be reduced by more than 50% if the carcasses do not exceed a limit of 1000 colony-forming units per gramme (cfu/g) (in the neck skin) [3]. The regulation stipulates that, since 1 January 2020, provided that no more than 15 pools out of 50 exceed 1000 cfu/g of *Campylobacter*, the process hygiene in the slaughterhouse is considered satisfactory. As of 1 January 2025, a maximum of 10 pools out of 50 only should exceed 1000 cfu/g *Campylobacter*.

About 55,486,000 broilers were counted in the agricultural census in Lower Saxony (LS) in 2020, which accounts for 60% of the total broiler chicken population in Germany [21]. Poultry meat production in Germany increased by 1.9% to 1,613,600 million tonnes in 2020 compared to that of 2019, and chicken meat production increased by 2.9% to 1,066,500 million tonnes compared to that of 2019 [22]. In this context, 539,428 tonnes of chicken meat were produced in LS in 2020 [23]. This is an increase of 5.0% compared to 2019 and accounts for about 50% of the total amount of produced chicken meat in Germany [22]. With the poultry meat production and the associated fattening poultry farming playing a significant role in Germany, especially in the Northwest of Germany, the neck samples of chicken carcasses of the PHC for *Campylobacter* of different slaughter lines in Northwest Germany were analysed in this study.

The research project analysed how *Campylobacter* contamination levels on neck skin samples of chicken carcasses at slaughterhouses in Northwest Germany and reported *Campylobacter* enteritis cases in Northwest Germany, Lower Saxony (LS) and North Rhine-Westphalia (NRW), that have evolved in recent years, especially before and during the COVID-19 pandemic. One aim of this study was to evaluate the correlation between *Campylobacter* levels in broilers and human cases in Northwest Germany.

## 2. Material and Methods

### 2.1. Campylobacter Levels in Broilers (PHC)

According to Regulation (EC) No. 2017/1495, slaughterhouses in Germany have been required since 1 January 2018 to test neck skin samples of chicken carcasses for *Campylobacter*. At least 15 neck skins of chilled carcasses must be sampled randomly at each sampling event. The sampling takes place weekly. The day of sampling has to be changed weekly. Before a microbiological examination in the laboratory, the neck skin samples from at least three chicken carcasses from the same flock of origin have to be pooled into one sample with a weight of 26 g. This results in a total of 5 × 26 g samples for the examination in the laboratory, as the 15 neck skin samples should weigh at least 130 g. The analytical reference method in the laboratory is ISO 10272-2 [24]. Each slaughterhouse thus has five weekly quantitative microbiological results on the occurrence of *Campylobacter* available since 1 January 2018. Data from several slaughter lines in Northwest Germany from 1 January 2018 to 31 December 2021 were included within the scope of this research project. The contamination levels of all slaughter lines were classified into three different categories: category one (C1), 0–99 cfu/g; category two, (C2) 100–999 cfu/g; and category three (C3), >1000 cfu/g.

### 2.2. Data on Human Campylobacter Cases

The laboratory detection of *Campylobacter* enteritis in Germany is notifiable in accordance with § 7 of the Protection Against Infection Act. Laboratory evidence is reported by the laboratory to the responsible local health department and from there transmitted anonymously via the state health department to the Robert Koch Institute (RKI), the Federal Public Health Institute. This study analysed the national surveillance data on notified *Campylobacter* infection cases for Northwest Germany (LS and NRW) from 2018 to 2021. Only cases fulfilling the reference definition were included, i.e., those cases with the clinical symptoms of *Campylobacter* with either a laboratory or epidemiologically confirmed *Campylobacter* infection. The data are publicly available via SurvStat@RKI 2.0 [25,26].

### 2.3. Methodology

Data on *Campylobacter* levels in broilers were summarised for a comparative illustration of each year and divided into time periods within a year. A time period extended over four calendar weeks. A calendar year began with time period 1 from week one to four, followed by time period 2 from week five to eight, etc., and the calendar year ended with time period 13 from week 49 to week 52. Thus, this amounted to 13 time periods per calendar year. A classification into months was not feasible. Because according to Regulation (EC) No. 2017/1495, the day of sampling has to be changed weekly [16]. The *Campylobacter* data from the slaughterhouses was provided per calendar week. It was therefore not possible to conclude the exact day on which the samples were taken. With this classification using time periods of four calendar weeks, we were able to ensure the reliable processing of the raw data. 

Category three (C3) neck samples of chicken carcasses (>1000 cfu/g *Campylobacter*) were used for the correlation analysis between *Campylobacter* levels in broilers and *Campylobacter* enteritis cases in Northwest Germany.

### 2.4. Statistical Analysis

Data analysis was performed using the statistical software package from SAS, Version 7.1 (SAS Inst., Cary, NC, USA). *Campylobacter* levels in broilers were analysed descriptively by mean values and minimum, maximum and standard deviation. To test for the normal distribution, a Shapiro–Wilk test was performed. Data were checked for significant differences with the Kruskal–Wallis test (one-way ANOVA) and additionally between single parameters using a post hoc test. Furthermore, the correlation between *Campylobacter* levels in broilers and *Campylobacter* enteritis cases was calculated using the Spearman’s rank correlation test. All statements of statistical significance were based on *p* < 0.05.

## 3. Results

### 3.1. Campylobacter Levels in Broilers

In the following section, the microbiological results of the neck samples of chicken carcasses according to PHC for *Campylobacter* of all slaughter lines are presented.

#### 3.1.1. *Campylobacter* Levels in Broilers per Year

The proportion of chicken carcass neck samples based on *Campylobacter* contamination levels for each category (C1, C2, C3) from 2018 to 2021 are shown in Table 1. The proportion of category three *Campylobacter* contamination levels changed significantly with the years. There was a significantly lower number of neck samples with *Campylobacter* levels above 1000 cfu/g in 2020 and 2021 (*p* < 0.05).

#### 3.1.2. *Campylobacter* Levels in Broilers per Time Period

**Figure 1:**
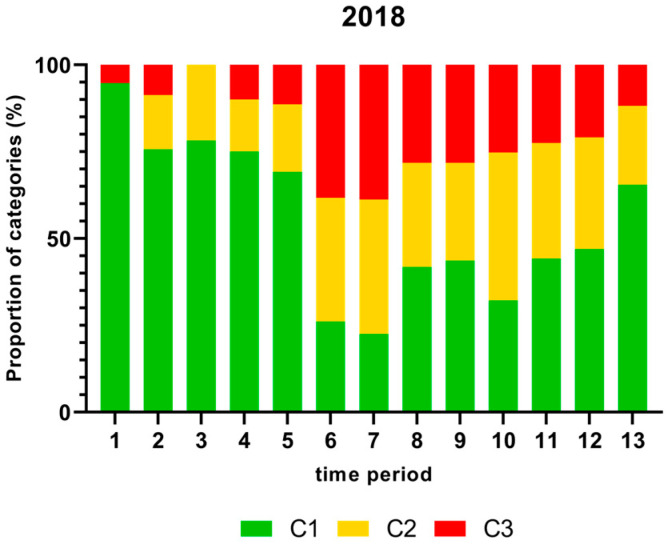
Proportion (%) of chicken carcass neck samples of all slaughter lines in accordance with the process hygiene criterion (PHC) for *Campylobacter* for each category (C1–C3) for every single time period in 2018. Category one (C1): 0–99 colony-forming units (cfu/g), category two (C2): 100–999 cfu/g, and category three (C3): >1000 cfu/g.

**Figure 2:**
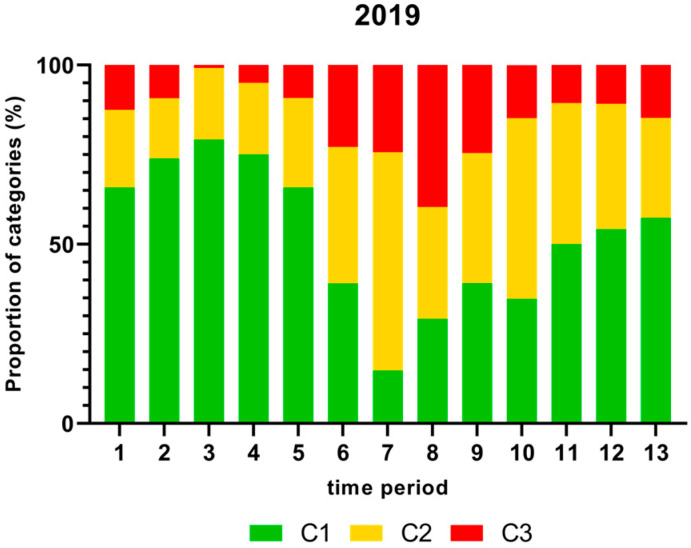
Proportion (%) of chicken carcass neck samples of all slaughter lines in accordance with the process hygiene criterion (PHC) for *Campylobacter* for each category (C1–C3) for every single time period in 2019. Category one (C1): 0–99 colony-forming units (cfu/g), category two (C2): 100–999 cfu/g, and category three (C3): >1000 cfu/g.

**Figure 3:**
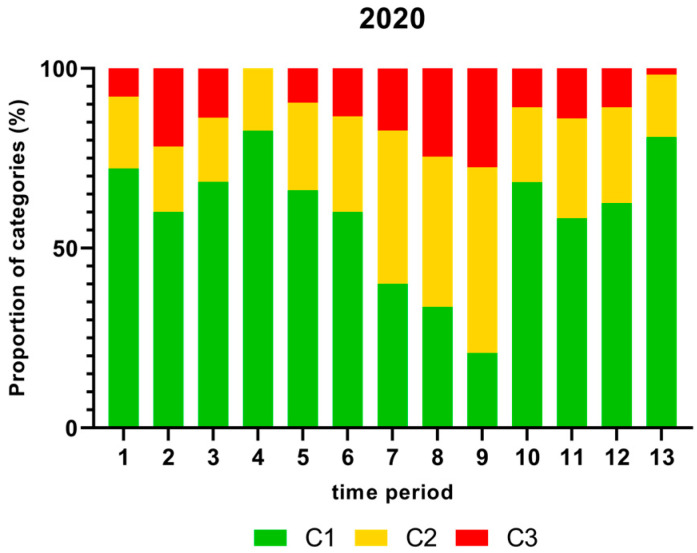
Proportion (%) of chicken carcass neck samples of all slaughter lines in accordance with the process hygiene criterion (PHC) for *Campylobacter* for each category (C1–C3) for every single time period in 2020. Category one (C1): 0–99 colony forming units (cfu/g), category two (C2): 100–999 cfu/g, and category three (C3): >1000 cfu/g.

**Figure 4:**
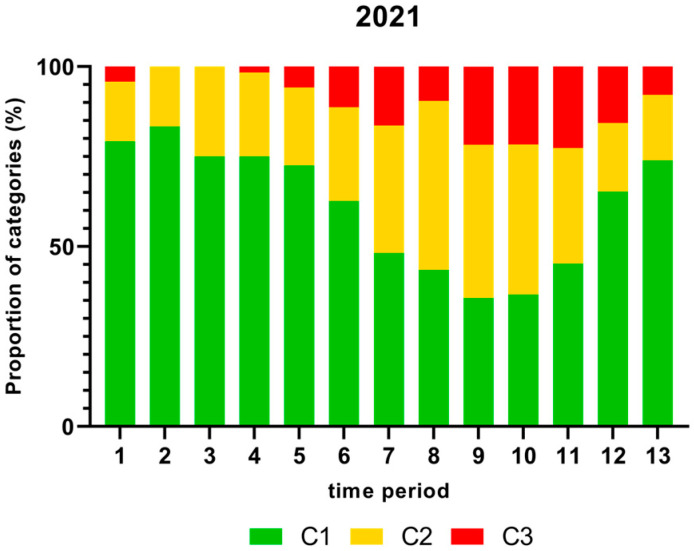
Proportion (%) of chicken carcass neck samples of all slaughter lines in accordance with the process hygiene criterion (PHC) for *Campylobacter* for each category (C1–C3) for every single time period in 2021. Category one (C1): 0–99 colony forming units (cfu/g), category two (C2): 100–999 cfu/g, and category three (C3): >1000 cfu/g.

Figure 1, Figure 2, Figure 3 and Figure 4 show the proportion of samples for each category for every single time period from 2018 to 2021. Each Figure shows the contamination levels of *Campylobacter* for one year. Additional statistical analyses are shown in Appendix A.

There was a significantly higher proportion (%) of chicken carcass neck samples containing more than 1000 cfu/g of *Campylobacter* in time periods 6 and 7 in 2018 and a significantly lower proportion in time period 3 as shown in Figure 1.

As shown in Figure 2, the proportion (%) of *Campylobacter* contamination levels on neck samples above 1000 cfu/g was significantly higher in time period 8 and lower in time periods 3 and 4 in 2019.

The highest proportion (%) of *Campylobacter* contamination levels on neck samples above 1000 cfu/g was shown in time period 9 and the lowest proportion in time period 4 in 2020 as displayed in Figure 3.

As displayed in Figure 4, the highest proportion (%) of *Campylobacter* contamination levels on neck samples above 1000 cfu/g was shown in time period 11 and the lowest in time periods 2 and 3 in 2021.

### 3.2. Data on Human Campylobacter Cases

*Campylobacter* enteritis cases in Northwest Germany, reported in accordance with the Protection Against Infection Act via SurvStat@RKI 2.0 [26], are displayed in the following section.

#### Number of Human Cases per Year

**Figure 5:**
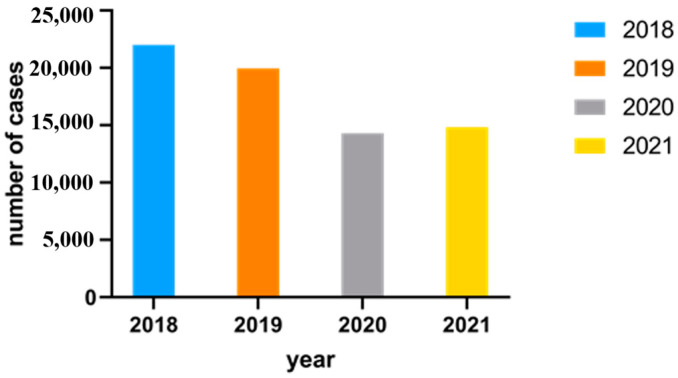
Number of *Campylobacter* enteritis cases in Northwest Germany from 2018 to 2021 via SurvStat@RKI 2.0.

Figure 5 displays and compares the number of *Campylobacter* enteritis cases in Northwest Germany from 2018 to 2021.

As illustrated in Figure 5, the highest number of cases were reported in 2018 (22,009 cases), and the least number of cases were reported in 2020 (14,299 cases).

### 3.3. Correlation between Campylobacter Levels in Broilers and Human Cases

#### Correlation between Category Three Campylobacter Contamination Levels on Chicken Carcass Neck Samples and *Campylobacter* Enteritis Cases in Northwest Germany from 2018 to 2021

Table 2 shows Spearman’s rank correlation between the number of chicken carcass neck samples containing >1000 cfu/g *Campylobacter* (C3) and human cases in Northwest Germany from 2018 to 2021. There was a higher positive correlation in 2018 and 2019. The correlation was lower in 2020 and 2021.

## 4. Discussion

### 4.1. Evaluation of Campylobacter Levels in Broilers

#### 4.1.1. Evaluation of Campylobacter Levels in Broilers per Year from 2018 to 2021

The aim of the PHC for *Campylobacter* was to control the contamination of carcasses during the slaughtering process and to reduce the load of *Campylobacter* on carcasses [16]. The mandatory testing in German slaughterhouses of broiler chickens as a part of the PHC for *Campylobacter*, which has been in force since January 2018, was used in this study to conduct an extensive analysis of the occurrence of *Campylobacter* at several slaughterhouses in Northwest Germany. Thus, the contamination levels of *Campylobacter* on chicken carcasses might have changed, since the beginning of testing in 2018.

The results show that the *Campylobacter* load and concentration from the examined neck skin samples decreased from 2018 to 2021 (Table 1). The proportion of chicken carcass neck samples of more than 1000 cfu/g decreased from an initial 19.40% in 2018 to almost half, i.e., 10.53%, in 2021 (Table 1). According to EFSA, this would be associated with a lower risk of *Campylobacter* infection for people who consume chicken meat [3].

In contrast to the microbiological findings of the PHC for *Campylobacter*, the results of investigations published by the Federal Office of Consumer Protection and Food Safety (BVL) in the zoonosis monitoring reports showed relatively constant values in recent years. The proportion of samples containing more than 1000 cfu/g was 22.6% in 2018, 23.4% in 2019, 21.9% in 2020 and 21.6% in 2021, respectively [28]. The reasons for differences between the results of the PHC for *Campylobacter* and the data published by the BVL are unclear. One possible reason could be that individual samples are required for zoonosis monitoring and pooled samples are required for PHC testing by food business operators in accordance with Regulation (EC) No. 2017/1495 [28].

The reduced contamination levels of *Campylobacter* on chicken carcasses at the slaughterhouse suggest that this results in a reduced *Campylobacter* contamination of retail chicken meat. However, the detection rate of *Campylobacter* in samples of fresh retail chicken meat in Germany shows consistent values in recent years. Prevalence testing showed that 47.8% of the samples were positive in 2018 and 46.4% thereof in 2019. In 2020, 54.7% of the retail samples tested positive and 46.9% thereof in 2021 [28]. These results highlight how frequently contaminated fresh chicken meat reaches consumers. The high prevalence of *Campylobacter* in fresh chicken meat was also published in the Netherlands and Denmark [29,30,31]. Despite the high prevalence in the samples of fresh retail chicken meat, many of these samples had quantitatively low levels of *Campylobacter*. Only 2.3% of the samples had *Campylobacter* levels above the detection limit of 10 cfu/g in 2020 [32]. In 2021, 2.8% of the samples contained more than 10 cfu/g *Campylobacter*. The high levels of *Campylobacter* (>1000 cfu/g) were not detected in retail in either year [28,32].

As *Campylobacter* contamination levels on neck samples have decreased in recent years, the results of our study suggest an increased awareness of *Campylobacter* as a foodborne pathogen both at the farm and the slaughterhouse level. High biosecurity measures are required on farms to prevent the introduction of *Campylobacter* into the flock and to protect flocks from colonisation [33]. Horvat et al. (2022) used a simulation model and showed that insect control had the strongest impact of all tested intervention measures to prevent *Campylobacter* contamination by reducing the percentage of highly contaminated (>1000 cfu/g) neck samples from 13% to 8% [34]. The low contamination levels of *Campylobacter* in broilers at primary production seem to have a major impact on reducing *Campylobacter* enteritis cases, as Foddai et al. (2022) investigated that targeted management measures on high-risk farms could significantly reduce the risk of *Campylobacter* infection for the consumer [35]. In organic or free-range farming, it is difficult to implement high biosecurity measures to prevent the introduction of *Campylobacter*. For this reason, organic broiler farms have a higher *Campylobacter* prevalence than conventional ones [36,37]. Cegar et al. (2022) showed that the presence of *Campylobacter* on chilled carcasses is more likely to be affected by their pre-slaughterhouse condition (at the farm level), rather than to be related to process hygiene at the slaughterhouse [38]. However, in addition to biosecurity at primary production, management measures at different stages of the slaughtering process are necessary to prevent the cross-contamination of *Campylobacter*, as Foddai et al. (2023) showed that reduced cross-contamination could minimise the risk of human *Campylobacter* cases efficiently [39]. A continuous survey of *Campylobacter* on farms does not exist. This would allow a better interpretation of intervention measures at this level. However, it can be concluded from the aforementioned studies that lower *Campylobacter* contamination levels on chicken carcass neck samples at the slaughterhouse are a result of the summation of efforts along the chicken meat production value chain.

#### 4.1.2. Evaluation of Campylobacter Levels in Broilers per Time Period from 2018 to 2021

Seasonal variations with a peak phase of *Campylobacter* in the summer months have been described in the literature [40]. In our study, higher contamination levels (>1000 cfu/g) were more present during the summer.

The proportion of chicken carcass neck samples that contained more than 1000 cfu/g *Campylobacter*, taking into account seasonal fluctuations, are shown in Figure 1, Figure 2, Figure 3 and Figure 4. In this study, many samples exceeded the limit of 1000 cfu/g *Campylobacter* during the summer in 2018 and 2019. A significantly higher percentage of neck samples peaked in time periods 6 and 7 in 2018, which refer to end of May until mid-July, and time period 8 in 2019 (mid-July to mid-August). In contrast, the proportion of samples containing more than 1000 cfu/g was lower in 2020 and 2021. The seasonal peaks during mid-summer in 2018 and 2019 are in agreement with results from the Netherlands with the highest contamination rate between June and September [41]. In contrast, in Norway, Kapperud et al. (1993) reported higher colonisation in late summer and autumn, with the highest colonisation from August to November [42]. The reasons for different seasonal peaks remain unclear, but can be related to climatic conditions [43,44].

### 4.2. Evaluation of Human Campylobacter Cases from 2018 to 2021

Regarding *Campylobacter* enteritis cases in Northwest Germany, a fewer number of cases were reported to the RKI in accordance with § 7 of the Protection Against Infection Act in recent years compared to 2018 (Figure 5). Thus, only 14,299 *Campylobacter* cases were reported in Northwest Germany in 2020. Slightly more *Campylobacter* cases were registered again in 2021. Reasons discussed for the reduced number of human cases in 2020 were the closure of restaurants, cafeterias, snack bars and canteens during the COVID-19 pandemic. As in addition to the consumption of chicken meat, eating out is the most important risk factor for *Campylobacter* infections [45]. There are other previous studies that show a risk factor in the consumption of chicken meat at restaurants [8,46,47,48]. In contrast, eating at home is considered protective or associated with a lower risk of infection with *Campylobacter* [46].

The lower risk of *Campylobacter* infection at home contrasts with results of other studies in which a poor hygienic behaviour in the own kitchen was repeatedly explained. Together with cross-contamination, it plays a significant role in the transmission of *Campylobacter* at home [49]. During the COVID-19 pandemic, hygiene measures to protect against the coronavirus, such as washing hands, cleaning and disinfection precautions, may have improved kitchen hygiene indirectly, thus breaking *Campylobacter* infection chains without the consumer being aware of it.

As a foodborne pathogen, people might have become more aware of the risk of *Campylobacter* infection in recent years and with this, fewer cases have occurred. However, this is contradicted by a survey conducted by the German Federal Institute for Risk Assessment (BfR) in August 2022, which states that many people are not aware of the pathogen *Campylobacter* and its association with foodborne infections [50]. Further studies comment on the poor awareness of consumers and their handling routine of fresh chicken meat [51,52].

### 4.3. Correlation between Campylobacter Levels in Broilers and Human Cases

The seasonality of human *Campylobacter* enteritis cases is well known and has been described in the literature [4,5,53]. Nylen et al. (2002) showed that seasonal peaks differed in European countries [54]. The seasonal peaks of high contamination levels on chicken carcass neck samples investigated in this study and the seasonality of human cases led to a correlation analysis. Fewer highly contaminated neck samples (>1000 cfu/g) and fewer human cases in recent years may have had an effect on the relationship between the two parameters. The results obtained in the current study revealed the highest correlation in 2018 (0.66) and the lowest correlation in 2020 (0.33) (Table 2). The lower correlation in 2020 and 2021 compared to 2018 and 2019 could indicate that the COVID-19 pandemic might have had an effect on the relationship between *Campylobacter* levels in broilers and human cases. However, this relationship requires more investigation, as the transmission routes of *Campylobacter* and especially the risk factors of chicken meat consumption could have been affected differently during the COVID-19 pandemic. With this in mind, it is relevant to examine to what extent the seasonality of human *Campylobacter* cases and the seasonality of *Campylobacter* in broilers are mutually dependent. Wei et al. (2015) showed that the prevalence of *Campylobacter* in broilers precedes *Campylobacter* incidence in humans [55]. In contrast, other studies described that the increase in human *Campylobacter* enteritis precedes the increase in broiler prevalence [56,57,58].

## 5. Conclusions

For the first time since the introduction of mandatory testing within the scope of the PHC for the presence of *Campylobacter* in slaughterhouses in Germany, data from several slaughter lines in Northwest Germany were analysed from 2018 to 2021. The results show that the proportion of neck samples with bacterial counts of more than 1000 cfu/g *Campylobacter* dropped continuously to almost half from 19.40% in 2018 to 10.53% in 2021. Thus, the limits of the PHC may have increased the awareness of *Campylobacter* as a foodborne pathogen in primary production and during the slaughtering process. A higher proportion of neck samples exceeded the limit of 1000 cfu/g *Campylobacter* during mid-summer. Correlation analysis between highly contaminated chicken carcass neck samples (>1000 cfu/g) and human cases showed a higher correlation in 2018 and 2019 prior to the COVID-19 pandemic. As a lower number of human cases in Northwest Germany was reported in 2020 and 2021 compared to 2018 and 2019, it remains uncertain whether this was due to the reduction in *Campylobacter* levels in relation to the PHC in slaughterhouses since 2018 or underreporting during the COVID-19 pandemic. As measures by the Federal State Governments were enacted to prevent a rapid spread of the coronavirus, they might have had an effect on the infection chain of *Campylobacter*. Whether there is a relationship between microbiological slaughter findings and the number of *Campylobacter* cases including seasonal fluctuations and if there is a causal connection between a decrease in *Campylobacter* levels in the slaughterhouse and among consumers in recent years have to be analysed in future studies.

## Figures and Tables

**Table 1:** Proportion (%) of chicken carcass neck samples of all slaughter lines in accordance with the process hygiene criterion (PHC) for *Campylobacter*, for each category (C1–C3) from 2018 to 2021. Category one (C1): 0–99 colony-forming units (cfu/g), category two (C2): 100–999 cfu/g, and category three (C3): >1000 cfu/g.

Year	C1	C2	C3
2018	54.44 ^AB^ ± 46.99	26.16 ^A^ ± 37.19	19.40 ^B^ ± 34.63
2019	52.49 ^A^ ± 47.25	32.30 ^A^ ± 40.17	15.20 ^AB^ ± 29.73
2020	59.46 ^AB^ ± 46	27.27 ^A^ ± 39.06	13.27 ^A^ ± 28.86
2021	61.45 ^B^ ± 45.3	28.03 ^A^ ± 39.22	10.53 ^A^ ± 25.22

^A,B^ means in a column with different superscripts differ significantly (*p* < 0.05).

**Table 2:** Relationship between the number of neck samples with *Campylobacter* contamination levels of >1000 cfu/g (C3) and *Campylobacter* enteritis cases in Northwest Germany from 2018 to 2021 according to Spearman’s rank correlation coefficient (SCC).

	2018	2019	2020	2021
SCC	0.66	0.58	0.33	0.45
*p*-value	<0.0001	<0.0001	0.0185	0.0009

Correlation levels classification: 0.0–0.3, “inexistent correlation”, 0.3–0.5, “weak positive correlation”, 0.5–0.7, “moderate positive correlation”, 0.7–0.9, “strong positive correlation”, and 0.9–1, “very strong positive correlation [27].

## Data Availability

Data is contained within the article.

## Appendix A

**Table A1:** Proportion (%) of chicken carcass neck samples of all slaughter lines in accordance with the process hygiene criterion (PHC) for *Campylobacter* for each category (C1–C3) for every single time period in 2018. Category one (C1): 0–99 colony forming units (cfu/g), category two (C2): 100–999 cfu/g, and category three (C3): >1000 cfu/g.

Time Period	C1	C2	C3
1	94.74 ^E^ ± 22.94	0.00 ^A^ ± 0.00	5.26 ^AB^ ± 22.94
2	75.65 ^DE^ ± 41.76	15.65 ^AB^ ± 34.62	8.70 ^ABC^ ± 28.81
3	78.18 ^DE^ ± 39.96	21.82 ^ABCD^ ± 39.96	0.00 ^A^ ± 0.00
4	75.00 ^DE^ ± 44.23	15.00 ^AB^ ± 35.02	10.00 ^ABC^ ± 28.89
5	69.17 ^CDE^ ± 44.13	19.44 ^ABC^ ± 35.98	11.39 ^ABC^ ± 28.59
6	26.09 ^A^ ± 38.35	35.65 ^BCD^ ± 36.67	38.26 ^D^ ± 40.41
7	22.50 ^A^ ± 36.50	38.75 ^CD^ ± 34.93	38.75 ^D^ ± 39.16
8	41.82 ^AB^ ± 46.56	30.00 ^BCD^ ± 38.91	28.18 ^CD^ ± 38.87
9	43.64 ^AB^ ± 46.04	28.18 ^BCD^ ± 35.27	28.18 ^CD^ ± 38.38
10	32.17 ^A^ ± 43.38	42.61 ^D^ ± 39.22	25.22 ^BCD^ ± 36.29
11	44.17 ^AB^ ± 47.54	33.33 ^BCD^ ± 40.29	22.50 ^BCD^ ± 37.45
12	46.96 ^ABC^ ± 47.71	32.17 ^BCD^ ± 39.88	20.87 ^BCD^ ± 36.92
13	65.45 ^BCD^ ± 46.67	22.73 ^BCD^ ± 39.18	11.82 ^ABC^ ± 31.26

^A–E^ means in a column with different superscripts differ significantly (*p* < 0.05).

**Table A2:** Proportion (%) of chicken carcass neck samples of all slaughter lines in accordance with the process hygiene criterion (PHC) for *Campylobacter* for each category (C1–C3) for every single time period in 2019. Category one (C1): 0–99 colony forming units (cfu/g), category two (C2): 100–999 cfu/g, and category three (C3): >1000 cfu/g.

Time Period	C1	C2	C3
1	65.83 ^DE^ ± 47.72	21.67 ^A^ ± 41.25	12.50 ^AB^ ± 33.78
2	73.91 ^DE^ ± 44.90	16.81 ^A^ ± 35.89	9.28 ^AB^ ± 26.57
3	79.17 ^E^ ± 41.49	20.00 ^A^ ± 40.00	0.83 ^A^ ± 4.08
4	75.00 ^DE^ ± 44.23	20.00 ^A^ ± 38.67	5.00 ^A^ ± 17.94
5	65.83 ^DE^ ± 47.72	25.00 ^A^ ± 40.97	9.17 ^AB^ ± 25.69
6	39.05 ^ABC^ ± 45.38	38.10 ^ABC^ ± 37.37	22.86 ^BC^ ± 31.80
7	14.78 ^A^ ± 27.11	60.87 ^C^ ± 33.29	24.35 ^BC^ ± 28.89
8	29.17 ^AB^ ± 40.85	31.25 ^AB^ ± 35.05	39.58 ^C^ ± 38.95
9	39.09 ^ABC^ ± 43.41	36.36 ^AB^ ± 39.83	24.55 ^BC^ ± 39.00
10	34.78 ^ABC^ ± 46.01	50.43 ^BC^ ± 39.94	14.78 ^AB^ ± 21.92
11	50.00 ^BCD^ ± 47.31	39.39 ^ABC^ ± 41.81	10.61 ^AB^ ± 24.92
12	54.17 ^BCDE^ ± 45.48	35.00 ^AB^ ± 41.39	10.83 ^AB^ ± 28.27
13	57.39 ^CDE^ ± 47.60	27.83 ^AB^ ± 40.33	14.78 ^AB^ ± 32.60

^A–E^ means in a column with different superscripts differ significantly (*p* < 0.05).

**Table A3:** Proportion (%) of chicken carcass neck samples of all slaughter lines in accordance with the process hygiene criterion (PHC) for *Campylobacter* for each category (C1–C3) for every single time period in 2020. Category one (C1): 0–99 colony forming units (cfu/g), category two (C2): 100–999 cfu/g, and category three (C3): >1000 cfu/g.

Time Period	C1	C2	C3
1	72.17 ^D^ ± 42.95	20.00 ^AB^ ± 36.68	7.83 ^ABC^ ± 26.10
2	60.00 ^CD^ ± 49.36	18.26 ^A^ ± 38.57	21.74 ^CDE^ ± 42.17
3	68.42 ^D^ ± 47.76	17.89 ^A^ ± 37.65	13.68 ^ABCDE^ ± 33.37
4	82.61 ^D^ ± 38.76	17.39 ^A^ ± 38.76	0.00 ^A^ ± 0.00
5	66.09 ^D^ ± 44.08	24.35 ^ABC^ ± 38.59	9.57 ^ABCD^ ± 26.19
6	60.00 ^CD^ ± 47.18	26.67 ^ABC^ ± 38.52	13.33 ^ABCDE^ ± 28.08
7	40.00 ^ABC^ ± 44.51	42.73 ^CD^ ± 39.66	17.27 ^BCDE^ ± 30.42
8	33.64 ^AB^ ± 45.10	41.82 ^BCD^ ± 40.43	24.55 ^DE^ ± 33.77
9	20.83 ^A^ ± 30.92	51.67 ^D^ ± 36.32	27.50 ^E^ ± 33.78
10	68.33 ^D^ ± 43.31	20.83 ^ABC^ ± 37.06	10.83 ^ABCD^ ± 27.65
11	58.26 ^BCD^ ± 44.28	27.83 ^ABC^ ± 39.88	13.91 ^ABCDE^ ± 28.56
12	62.50 ^CD^ ± 46.18	26.67 ^ABC^ ± 40.29	10.83 ^ABCD^ ± 25.01
13	80.87 ^D^ ± 38.37	17.39 ^A^ ± 35.32	1.74 ^AB^ ± 8.34

^A–E^ means in a column with different superscripts differ significantly (*p* < 0.05).

**Table A4:** Proportion (%) of chicken carcass neck samples of all slaughter lines in accordance with the process hygiene criterion (PHC) for *Campylobacter* for each category (C1–C3) for every single time period in 2021. Category one (C1): 0–99 colony forming units (cfu/g), category two (C2): 100–999 cfu/g, and category three (C3): >1000 cfu/g.

Time Period	C1	C2	C3
1	79.17 ^D^ ± 41.49	16.67 ^A^ ± 38.07	4.17 ^ABC^ ± 20.41
2	83.33 ^D^ ± 38.07	16.67 ^A^ ± 38.07	0.00 ^A^ ± 0.00
3	75.00 ^D^ ± 41.39	25.00 ^ABC^ ± 41.39	0.00 ^A^ ± 0.00
4	75.00 ^D^ ± 40.97	23.33 ^AB^ ± 39.42	1.67 ^AB^ ± 8.16
5	72.50 ^CD^ ± 40.78	21.67 ^AB^ ± 36.32	5.83 ^ABC^ ± 21.65
6	62.61 ^BCD^ ± 47.60	26.09 ^ABC^ ± 39.28	11.30 ^ABCDE^ ± 27.52
7	48.18 ^ABC^ ± 45.21	35.45 ^ABC^ ± 38.01	16.36 ^CDE^ ± 22.79
8	43.48 ^AB^ ± 42.92	46.96 ^C^ ± 38.90	9.57 ^ABCDE^ ± 21.63
9	35.65 ^A^ ± 42.62	42.61 ^BC^ ± 38.76	21.74 ^DE^ ± 28.87
10	36.67 ^A^ ± 41.56	41.67 ^BC^ ± 38.64	21.67 ^DE^ ± 32.26
11	45.22 ^AB^ ± 44.81	32.17 ^ABC^ ± 40.33	22.61 ^E^ ± 36.33
12	65.22 ^BCD^ ± 47.18	19.13 ^A^ ± 36.42	15.65 ^BCDE^ ± 35.14
13	73.91 ^D^ ± 44.90	18.26 ^A^ ± 38.57	7.83 ^ABCD^ ± 26.10

^A–E^ means in a column with different superscripts differ significantly (*p* < 0.05).
